# Association between grandparent co-residence, socioeconomic status and dental caries among early school-aged children in Japan: A population-based prospective study

**DOI:** 10.1038/s41598-019-47730-3

**Published:** 2019-08-05

**Authors:** Ayako Morita, Yusuke Matsuyama, Aya Isumi, Satomi Doi, Manami Ochi, Takeo Fujiwara

**Affiliations:** 1 Department of Global Health Promotion, Tokyo Medical and Dental University, 1-5-45 Yushima, Bunkyo-ku, Tokyo 113-8510 Japan; 2 Department of Health and Welfare Services, National Institute of Public Health, 2-3-6 Minami, Wako-shi, Saitama 351-0197 Japan

**Keywords:** Risk factors, Oral diseases

## Abstract

Globally many children are living with grandparents, and it has been suggested that grandparent co-residence may be associated with dental caries in infants and toddlers possibly through passive parenting style, accompanied by children’s cariogenic behaviors such as feeding sugary sweets. However, little is known about this association in schoolchildren, adjusted for socioeconomic status. Therefore, this study investigates the association between grandparent co-residence, socioeconomic status, and dental caries among schoolchildren. All caregivers of first-grade children (age 6–7 years) in Adachi City, Tokyo, were administered a questionnaire about children’s grandparent co-residence status and oral health-related behaviors, and responses were linked with dental examination records conducted by school dentists (N = 3,578). Multilevel Poisson regression analysis was applied to examine the association between grandparent co-residence, socioeconomic status, and dental caries status for each individual tooth, adjusting for potential covariates. The percentage of dental caries experience was higher among children living with grandparents (48.9%) than among children living without grandparents (44.0%). The risk for caries, however, did not differ according to grandparent co-residence status when tooth type, child’s age and sex, and parental socio-economic status and structure were adjusted (PR, 1.13; 95%CI, 0.90, 1.42). The association between grandparent co-residence and dental caries among early school-aged children in urban Japan was confounded by socioeconomic status.

## Introduction

Dental caries is the most widespread chronic disease, affecting a vast majority of schoolchildren and nearly all adults in most industrialized countries^1^. In Japan, the most recent national school surveillance data indicate that approximately half of primary schoolchildren have one or more dental caries^2^. Dental caries can have serious and lasting complications such as pain and tooth loss, as well as reduction in children’s abilities to eat, speak and learn^3^. In light of dental caries prevention, it is important to initiate interventions to prevent caries at an early age. Identifying the key risk factors is among the first steps towards developing an effective intervention.

It is well-known that one of the determinants of dental caries among children is socioeconomic environments^4^. To date, research has largely focused on parents as they play the major role in dietary and oral hygiene behavioural acquisition in young children^5^. The study showed that parental socio-economic status, such as low maternal educational level and low household income level, and family structure, such as family size, single parent, and presence of an older sibling at home, are associated with increased risk of dental caries development^6–13^. In line with this endeavour of the association between social environment and paediatric dental caries, several studies reported that children aged 1.6 years old and 3 years old living with or looked after by grandparents were more likely to have dental caries^14–17^ particularly in urban children^18^. However, the results are not consistent in older children and few studies have adjusted for parental socioeconomic status, which might confound the association between grandparent co-residence and dental caries of children.

In recent years, research interest has grown towards the role of grandparents in dental caries development among children as a primary or a secondary caregiver^19,20^. Today, grandparent co-residence is common not only in developing countries but also worldwide (e.g., 12% in the United States in 2015^21^, and 20% in Japan in 2016^22^). Grandparents are more involved in childrearing, not just because of extended longevity but also due to family structural changes (e.g., rises in lone-parent households) and socioeconomic trends (e.g., increases in financial difficulties)^23–25^. It was reported that grandparents hold poor knowledge of nutritional and oral hygiene requirements for children^26,27^, and children aged 1.6 years old and 3 years old living with or looked after by grandparents have been suggested to have increased risk of dental caries^14–17^, particularly in urban children^18^. However, the results are not always consistent in 4–5 year-old children^16^, and the effects in children who are more independent in self-care and whose deciduous teeth start to shed while permanent teeth start to grow (around 6–7 years old) are unknown. Also, previous studies that compared the risk of dental caries between children who live with grandparents and those who do not did not adjust for the effects of fragile family socio-economic and structural characteristics, which are important confounding factors, and hence making it difficult to interpret the results.

The Adachi Child Health Impact of Living Difficulty (A-CHILD) study^28–33^ is a population-based prospective cohort data of children in Adachi City, Tokyo, Japan. A-CHILD study has information on health and living conditions, including detailed clinical oral data assessed by school dentists and family socio-economic characteristics and structure of school-aged children. The present study uses the A-CHILD study data and examines the association of grandparent co-residence, parental socioeconomic status, dental caries of children in a prospective cohort of first-grade Japanese children.

## Results

Table 1 shows the characteristics of the sample. The percentage of the children living with grandparents was 9.3%. Overall, 44.4% of the participants had at least one dental caries with the mean (SD) of 1.68 (2.58), with a range from 0 to 14 for dft and 0–4 for DMFT, in the second-grade (the distributions of dft and DMFT are shown in Supplementary Fig. 1). The percentage of dental caries experience was higher among children living with grandparents (48.9%) than among children living without grandparents (44.0%). Demographic and family characteristics of children who live with and who do not live with grandparents are presented in Table 1. Compared with children who do not live with grandparents, children who do were more likely to live in households with lone-parent, mothers with lower educational levels and lower income levels. There was no difference between them for child’s sex, age in months, and birth order.

**Table 1:** Baseline demographics and family characteristics of the participants by grandparent co-residence (n = 3,578).

	All (n = 3,578)		Grandparent co-residence				*p*
			No (n = 3,247)		Yes (n = 331)		
	n	%	n	%	n	%	
Sex							0.48
Male	1,814	50.7	1,640	50.5	174	52.6	
Female	1,754	49.0	1,597	49.2	157	47.4	
Unknown	10	0.3	10	0.3	0	0.0	
Age (month)	85.2	2.4	85.2	2.6	85.2	25.7	0.71
Living arrangement with parents							<0.001
Living with two parents	3,265	91.3	3,020	93.0	245	74.0	
Lone-parent household	310	8.7	224	6.9	86	26.0	
Missing	3	0.1	3	0.1	0	0.0	
Maternal education							<0.001
Highschool graduate or less	1,235	34.5	1,093	33.7	142	42.9	
Some college	1,504	42.0	1,379	42.5	125	37.8	
College or University graduate	753	21.0	703	21.7	50	15.1	
Other	23	0.6	17	0.5	6	1.8	
Missing	63	1.8	55	1.7	8	2.4	
Household income (yen)							0.010
<3 million	376	10.5	329	10.1	47	14.2	
3 million ~ <6 million	1,434	40.1	1,309	40.3	125	37.8	
6 million ~ <10 million	1,118	31.2	1,028	31.7	90	27.2	
10 million and above	310	8.7	285	8.8	25	7.6	
Missing	340	9.5	296	9.1	44	13.3	
Birth order							0.12
Middle or last-born	1,896	53.0	1,734	53.4	162	48.9	
First-born or only child	1,682	47.0	1,513	46.6	169	51.1	

*p* value is derived from Chi-square test for all except where at least one column had less than 10 samples, then Fisher’s exact test as performed.

Table 2 presents the comparison of dietary and oral hygiene behaviours by grandparent co-residence status. Children who live with grandparents received less parental control over snack intake and supervised tooth brushing practice and less frequently brushed tooth while consumed more sugar-sweetened beverages than those who do not live with grandparents. After adjustment for demographic and family characteristics, prevalence of parental control over snack intake were lower for children who live with grandparents than for those who do not live with grandparents (adjusted PR: 0.74, 95%CI: 0.63, 0.87). However, there was no significant differences between the groups with respect to prevalence of daily sugar-sweetened beverage intake (adjusted PR: 1.09, 95%CI: 0.87, 1.37), frequency of tooth brushing (adjusted PR: 0.96, 95%CI: 0.90, 1.04) and supervised tooth brushing practice (adjusted PR: 0.99, 95%CI: 0.93, 1.04).

**Table 2:** Prevalence and adjusted prevalence ratios (APR) and 95% confidence intervals (95%CI) for dietary and oral-hygiene behaviors in children by co-residence with grandparents (n = 3,578).

	Parental control over snack intake (not given/eat at certain time)			Sugar-sweetened beverage intake (≥1 time/day)			Tooth brushing (twice or more/day)			Supervised tooth brushing practice (yes)		
	%	APR	95%CI	%	APR	95%CI	%	APR	95%CI	%	APR	95%CI
**Grandparent co-residence**												
Yes	63.2	0.74	(0.63–0.87)	25.4	1.09	(0.87–1.37)	71.9	0.96	(0.90–1.04)	82.2	0.99	(0.93–1.04)
No	74.5	ref		20.1	ref		77.1	ref		85.8	ref	
Child’s age (months)		1.00	(0.98–1.01)		0.99	(0.97–1.01)		1.00	(0.99–1.01)		0.99	(0.99–1.00)
**Child’s sex**												
Male	72.7	ref		21.5	ref		75.3	ref		86.4	ref	
Female	74.3	1.09	(0.97–1.22)	19.7	0.90	(0.78–1.03)	77.9	1.04	(1.00–1.07)	84.4	0.98	(0.96–1.01)
**Mother’s educational attainment**												
Highschool graduate or less	64.3	ref		27.8	ref		73.4	ref		81.3	ref	
Some college	77.2	1.51	(1.33–1.72)	18.3	0.70	(0.60–0.81)	79.2	1.06	(1.02–1.11)	87.2	1.05	(1.01–1.08)
College or University graduate	82.1	1.94	(1.60–2.35)	13.2	0.53	(0.42–0.66)	77.4	1.04	(0.98–1.09)	88.8	1.05	(1.01–1.09)
Other	79.0	2.15	(0.81–5.67)	15.8	0.64	(0.23–1.74)	60.9	0.85	(0.61–1.19)	90.9	1.10	(0.96–1.27)
**Household economic status**												
<3 million	67.3	ref		26.2	ref		72.1	ref		80.1	ref	
3 million ~ <6 million	73.9	1.27	(1.03–1.55)	22.5	0.88	(0.69–1.12)	75.8	1.03	(0.95–1.11)	84.5	1.02	(0.96–1.09)
6 million ~ <10 million	77.3	1.32	(1.06–1.64)	16.5	0.73	(0.56–0.95)	79.9	1.08	(0.99–1.17)	87.4	1.05	(0.99–1.11)
10 million and above	74.3	1.09	(0.82–1.45)	15.6	0.73	(0.51–1.06)	79.7	1.08	(0.98–1.19)	91.3	1.10	(1.03–1.17)
**Living arrangement with parents**												
Lone-parent household	73.9	1.10	(0.89–1.37)	20.1	1.01	(0.78–1.31)	77.1	0.96	(0.87–1.04)	86.4	0.92	(0.86–0.99)
Living with both parents	68.1	ref		26.1	ref		70.9	ref		76.1	ref	
**Birth order**												
First-born or only child	77.2	ref		19.8	ref		79.5	ref		89.7	ref	
Middle or last-born	69.3	0.79	(0.71–0.89)	21.5	1.03	(0.90–1.18)	73.3	0.92	(0.89–0.96)	80.7	0.90	(0.88–0.93)

APR = Adjusted Prevalence Ratio derived from a model that included all the variables presented in the table (i.e., grandparent co-residence, age, sex, birth order, living arrangement with parents, mother’s educational attainment and household economic status).

Table 3 presents the status of each tooth (N of participants = 3,578; n of teeth = 80,897). There were more deciduous teeth (n of individual teeth = 48,753) than permanent teeth (n of individual teeth = 32,326). Dental caries prevalence varied by tooth type and position. It was more prevalent in deciduous teeth (11.6%) than in permanent teeth (1.1%) and more prevalent in back teeth (e.g., 24.6% in lower first molars in deciduous teeth/first premolars in permanent teeth; 18.6% in lower second molars in deciduous teeth/first premolars in permanent teeth; 15.9% in upper first molars in deciduous teeth/first premolars in permanent teeth; 14.0% in lower first molars in deciduous teeth/first premolars in permanent teeth) than in front teeth (e.g., 1.6% in upper central and lateral incisors; 0% in lower central incisors, 0.1% in lower lateral incisors). The status of each tooth by grandparent co-residence is shown in Supplementary Table 1.

**Table 3:** Outcome prevalence of teeth by tooth type and position (n of individual teeth [level 1] = 80,897; n [level 2] = 3,578).

	All teeth		Decidious teeth		Permanent teeth		Duplication of deciduous tooth and its permanent successor in the same position
	decayed or filled/decayed, missing or filled	Sound	decayed or filled	Sound	decayed, missing or filled	Sound	
All positions	5,988	74,909	5,631	43,122	358	31,967	181
Upper central incisor	105	6,455	100	1,192	5	5,294	31
Upper lateral incisor	95	5,737	92	3,854	3	1,906	23
Upper canine	110	6,912	110	6,906	0	7	1
Upper first molar in deciduous teeth/first premolar in permanent teeth	1,130	5,969	1,129	5,910	1	64	5
Upper second molar in deciduous teeth/second premolar in permanent teeth	993	6,119	993	6,112	0	11	4
Upper first molar in permanent teeth	116	5898	—	—	116	5898	—
Upper second molar in permanent teeth	0	0	—	—	0	0	—
Lower central incisor	0	7,061	0	192	0	6,896	27
Lower laterlal incisor	9	6,590	6	1,221	4	5,441	73
Lower canine	141	6,684	141	6,604	0	89	9
Lower first molar in deciduous teeth/first premolar in permanent teeth	1,736	5,362	1,736	5,344	0	25	7
Lower second molar in deciduous teeth/second premolar in permanent teeth	1,324	5,791	1,324	5,787	0	5	1
Lower first molar in permanent teeth	229	6,331	—	—	229	6,331	—
Lower second molar in permanent teeth	0	0	—	—	0	0	—

Note: In total, 7 deciduous teeth was recorded as missig teeth due to caries but they were not coded as dental caries following the standard procedure of the school health checkups. In total, 181 cases were identified where deciduous tooth and its permanent successor were growing in the same position. Although 1 permanent successor was identified as decayed, missing or filled, it is likely that permanent successors were just grown and we dropped permanent successors from the analysis in Table 4 and not presented in all teeth category in this table.

Table 4 shows the results of multilevel analysis on the association between grandparent co-residence and caries status of each tooth (N of participants = 3,578, n of teeth = 80,897). The Interclass Correlation Coefficient (ICC) estimate showed approximately 60–70% of the variance can be attributed to variations between individuals, indicating that dental caries is highly likely to be developed by the same individual. Grandparent co-residence was positively associated with dft/DMFT in the second grade (Crude PR: 1.28, 95%CI: 1.03, 1.60); however, the positive association disappeared when further adjusted for parental SES (Adjusted PR: 1.15, 95%CI: 0.92, 1.45). Final model adjusted for the absence of parental control over snack intake and daily sugar-sweetened beverage intake and frequent tooth brushing remain to show non-significant association between grandparental co-residence and dental caries. Sensitivity analyses stratified by tooth type showed that the positive association between grandparent co-residence and caries among deciduous teeth also disappeared when adjusted for parental SES (Adjusted PR: 1.15, 95%CI: 0.92, 1.45) (Supplementary Table 2) and no association was observed between grandparent co-residence and caries among permanent teeth (Crude PR: 1.22, 95%CI: 0.72, 2.05) (Supplementary Table 3).

**Table 4:** Multilevel poisson regression analysis of dental caries experience (decayed or filled primary teeth and Decayed, Missing, or Filled permanent teeth) in the second grade (n of individual teeth [level 1] = 80,897; n [level 2] = 3,578) by grandparent co-residence.

Outcome	Crude		Model I		Model II		Model III		Model IV	
	PR	95%CI	APR	95%CI	APR	95%CI	APR	95%CI	APR	95%CI
**Grandparent co-residence**										
Yes	1.28	(1.03–1.60)	1.29	(1.03–1.61)	1.15	(0.92–1.45)	1.13	(0.90–1.42)	1.06	(0.85–1.33)
No	ref		ref		ref		ref		ref	
**Tooth type**										
Decidious tooth	ref		ref		ref		ref		ref	
Permanent tooth	0.09	(0.08–0.97)	0.09	(0.08–0.10)	0.09	(0.08–0.10)	0.09	(0.08–0.10)	0.09	(0.08–0.10)
**Sex**										
Male	ref		ref		ref		ref		ref	
Female	0.85	(0.74–0.97)	0.90	(0.78–1.03)	0.89	(0.78–1.02)	0.87	(0.76–0.99)	0.90	(0.78–1.02)
Age (month)	1.00	(0.98–1.02)	1.00	(0.98–1.02)	1.00	(0.98–1.02)	1.00	(0.98–1.02)	1.00	(0.98–1.02)
**Maternal education**										
Highschool graduate or less	ref				ref		ref		ref	
Some college	0.73	(0.63–0.85)			0.76	(0.66–0.89)	0.79	(0.68–0.92)	0.88	(0.76–1.03)
College or University graduate	0.50	(0.41–0.60)			0.53	(0.43–0.64)	0.57	(0.47–0.70)	0.67	(0.55–0.81)
Other	0.66	(0.28–1.56)			0.67	(0.28–1.60)	0.64	(0.27–1.53)	0.71	(0.30–1.69)
**Household income (yen)**										
<3 million	ref				ref		ref		ref	
3 million ~ <6 million	0.72	(0.58–0.90)			0.83	(0.65–1.06)	0.81	(0.64–1.04)	0.85	(0.66–1.08)
6 million ~ <10 million	0.51	(0.40–0.64)			0.63	(0.48–0.81)	0.61	(0.47–0.79)	0.66	(0.51–0.85)
10 million and above	0.63	(0.47–0.85)			0.87	(0.63–1.21)	0.82	(0.59–1.14)	0.89	(0.65–1.23)
**Living arrangement with parents**										
Living with two parents	ref				ref		ref		ref	
Lone-parent household	1.61	(1.29–2.01)			1.20	(0.92–1.56)	1.22	(0.94–1.58)	1.20	(0.93–1.56)
**Birth order**										
First-born or only child	ref						ref		ref	
Middle or last-born	1.73	(1.51–1.97)					1.69	(1.48–1.93)	1.58	(1.39–1.81)
**Parental contorl over snack intake**										
Conrolled by parent(s)	ref								ref	
Eating at any time	1.96	(1.69–2.28)							1.56	(1.33–1.82)
**Sugar-sweetened beverage intake**										
<1 time/day	ref								ref	
1 time or more/day	1.89	(1.61–2.22)							1.58	(1.34–1.86)
**Tooth brushing**										
<2 times/day	ref								ref	
2 times or more/day	0.64	(0.55–0.74)							0.79	(0.68–0.92)
**Supervised tooth brushing practice**										
No	ref								ref	
Yes	0.65	(0.55–0.78)							0.86	(0.71–1.03)

PR = Prevalence Ratio; APR = Adjusted Prevalence Ratio (Model I adjusted for tooth type and demographics; Model II further adjusted for parental SES; Model III further adjusted for birth order; Model IV further adjusted for health behaviors).

## Discussion

Our results showed that the association between grandparent co-residence and dental caries experience was explained by parental SES, namely, low maternal education and low household income. The lower parental SES contributed to the reduced prevalence of parental control over snack intake, frequent tooth-brushing and tooth-brushing supervision, the increased prevalence of daily sugar-sweetened beverage intake, and the increased risk of dental caries in the second-grade. To the best of our knowledge, this is the first population-based study which showed that the association between grandparent co-residence and dental caries experience in school-aged children could be confounded by their common cause, low parental SES. To utilize our unique data on tooth-level outcomes, we employed multilevel modelling and adjusted for tooth types and tooth positions that differ for dental caries risk.

Our findings are inconsistent with earlier studies that reported the association between grandparent co-residence and poor oral health related behaviours and higher risk of dental caries among Japanese children aged 1.6 and 3 years old^14–18^. Although it was expected that children living with grandparents would receive passive parenting from grandparents, and they would eat more snack, sweets, and less likely brush their teeth, we found that prevalence of daily sugary beverage intake, frequent tooth brushing and supervised tooth brushing practices did not differ by grandparent co-residence status. Children become more independent in their self-care and less dependent on their caregivers, and are more informed of the updated health recommendation at school. A previous study has reported significant association between grandparent co-residence and dental caries in 3-year-olds, whereas no association was found in 4-year-olds and 5-year-olds^16^. Thus, children may be less influenced by grandparents when children became older. Also, children start to replace deciduous teeth with permanent teeth around 6 years old^34^, which leads to alleviate early effects on dental caries development.

Our study strength lies into availability and adjustment of information about socioeconomic status and family structure. They have been identified as one of the major precursors for grandparent co-residence in modern societies after 1970s^35^ and well-known risk factors for dental caries^4,36–38^. In Japan, grandparent co-residence has been commonly initiated by filial piety. However, our data showed that it is associated with low SES and fragile family structure, which suggests that grandparent co-residence may be commonly initiated in response to family crisis such as divorce and economic difficulty in urban Japan today, as in West^39^. It is important to adjust for SES and family structure when examining the association between grandparent co-residence and dental caries in children.

Nonetheless, this study has limitations. First, we lacked data on who was providing food and tooth-brushing the children. Although grandparent co-residence reflects the opportunity structure that facilitates grandparent-grandchild interactions, children from nuclear families might have also received care from grandparents who live nearby leading to underestimation of the grandparents’ effects at home. Second, a majority of the questionnaires were filled out by parents, and it is possible that grandparents influenced on health behaviours of children during parental absence without being noticed. Third, our sample size limited statistical power to perform sub-group analysis among grandparent co-residence households such as by grandparents’ sex, age, health and working status, and relational characteristics (i.e., paternal or maternal). Future studies that directly investigate grandparent childcare and their influence on child health status in diverse family types are needed to understand the influence of grandparents on grandchildren’s health behaviours and health more. Finally, we examined the second-grade children who have just erupted the first molars and have not developed full sets of permanent teeth. As caries rises rapidly to the maximum rate approximately two to three years post-eruption^40^, we may have underestimated the effects of grandparent co-residence. Future studies investigating the older children are required before concluding the effects of grandparent co-residence on school-age children.

Although our result indicated that grandparent co-residence was not the “direct cause” of dental caries, the finding has important implication in dental caries prevention. Several caries prevention strategies such as fluoride mouthrinse in school^41^ and supporting dental sealant in dental clinic has been implemented in some municipalities; however, children with lower family SES are still more likely to develop dental caries^42^. An intervention study has shown that grandparents could be empowered as caregivers and contribute to decrease childhood behavioural problems^43^. For high-risk households, they are considered as a valuable resource to promote children’s health and well-beings by serving as role models and providing instrumental support to parents in the home setting^44^. Training and supervising grandparents with educational handbooks and classes about importance and strategies to prevent childhood dental caries could potentially reduce their grandchildren’s risk of dental caries. Future studies should be conducted with such interventions and compare dental caries experience of infants and children whose co-residing grandparents received the proposed intervention with a similar high-risk group.

In conclusion, our findings indicate that grandparent co-residence reflects low maternal education and low income, which are associated with the increased risk of dental caries in the early school-age children in urban Japan. The current findings can be useful information to consider effective interventions to promote dental caries prevention among families raising elementary school age children.

## Methods

We followed the STROBE guideline for the analysis of cohort data.

### Study sample

All the parents/guardians of the first-grade children attending public school in Adachi City, Tokyo, Japan (student n = 5,355; school n = 69) were invited to participate in the study in 2015 (response rate: 80.1%) and in 2016 for a follow-up (response rate: 81.4%). In total, 3,711 parents/guardians filled out the survey in both years and agreed to link their responses with school health data. We excluded children who did not participate in the school dental health check-ups in 2016 (n = 23) and children who changed grandparent co-residence status during the follow-up (n = 100), resulting in a final sample of 3,578 children. The study was approved by the research ethics committees at the National Centre for Child Health and Development, and Tokyo Medical and Dental University, and performed in accordance with the Declaration of Helsinki and the Japan’s Ethical Guidelines for Epidemiological Research established by the Ministry of Education, Culture, Sports, Science and Technology and the Ministry of Health, Labour and Welfare. All the caregivers (a parent or legal guardian) provided written informed consent for study participation.

### Measurements

Information about grandparent co-residence, which was defined as at least one grandparent is listed as a household member, was asked via the questionnaire in the first grade (6–7 years). Dietary and oral hygiene behaviours were also asked in the same questionnaire and the responses were dichotomized according to recommendations by the American Academy of Pediatric Dentistry^45,46^: (1) parental control over snack intake (letting the child eat freely vs. placing the time to eat/not giving at all), (2) sugar-sweetened beverage intake (2 times or more per day/1 time a day, vs. 4–6 times a week/2–3 times a week/once a week/a few times a month/not at all), (3) tooth brushing (twice or more per day vs. once a day/not every day/unknown) and (4) supervised tooth brushing practice (yes or no).

For the outcome, we evaluated dental caries status in the second grade (7–8 years) for individual tooth. School dentists examined all the children with a plane mouth mirror under standardized lighting in a classroom as a part of annual oral health examination conducted according to the School Health and Safety Act. The dentists followed the standard procedures and guidelines to record any visible caries^47^. We defined having dental caries as the deciduous tooth being decayed or filled (dft) or permanent tooth being decayed, missing or filled (DMFT) and determined the status for each tooth. If children having both deciduous teeth and permanent teeth in the same position, we used the permanent teeth status. With respect to tooth position, we classified teeth into 14 categories (i.e., upper central incisor, upper lateral incisor, upper canine, upper first molar in deciduous teeth/first premolar in permanent teeth, upper second molar in deciduous teeth/second premolar in permanent teeth, upper first molar in permanent teeth, upper second molar in permanent teeth, lower central incisor, lower lateral incisor, lower canine, lower first molar in deciduous teeth/first premolar in permanent teeth, lower second molar in deciduous teeth/second premolar in permanent teeth, lower first molar in permanent teeth, lower second molar in permanent teeth) according to Logan & Kronfeld^48^, assuming that caries risk between left and right side within upper or lower arches is equal^49^.

With respect to potential covariates, we measured basic demographics (child’s sex and age in months) and family structure (living arrangement with parents: dual-parent household vs. lone-parent household; birth order: middle or last-born vs. first-born or only child) from the same household composition question used to identify grandparent co-residence. Those who were not living with father or mother for reasons such as divorce, separation, decease, not married and living apart for work, were classified into one group as lone-parent household. We also measured family socio-economic status by maternal education (junior high school graduate, high school graduate, some college or college graduate, university graduate, other) and annual household income (presented 10 categories with JPY500,000 intervals, starting with <JPY500,000 and ending in JPY10,000,000 and over to the participants and set the lowest household income as less than JPY3,000,000 or USD20,235 as of March 18^th^, 2019).

### Statistical analysis

First, we compared the baseline demographic and family environmental characteristics of children who live with grandparents and those who do not, using t-test for continuous independent variables and chi-square test for categorical independent variables. Next, we calculated the prevalence ratio (PR) of dietary and oral hygiene behaviours in the first grade (6–7 years) by grandparent co-residence, using Poisson model and adjusted for the demographic and family environmental characteristics. Finally, we employed multilevel Poisson regression model to investigate whether grandparent co-residency is associated with dental caries status for each tooth (1 = decayed or filled teeth/Decayed, Missing or Filled Teeth, 0 = sound teeth) in the second grade (7–8 years), nested within individuals^50^. We chose this method over the a single-level analysis method because our study participants were in transition from primary to permanent dentition, thus the likelihood of dental caries experience is considerably affected by the existing teeth’s position within the mouth. We built crude and other four models where the first model adjusted for tooth type and demographics, the second model further adjusted for parental SES, the third model further adjusted for birth order and the final model further adjusted for health behaviours. The third and final models were built in order to explore the potential mechanisms behind the association between grandparent co-residence and dental caries. STATA 14.0 was used to perform all the analyses.

## Supplementary information

Supplementary figure and tables

## Data Availability

The datasets generated during and/or analyzed during the current study are available from the corresponding author on reasonable request.
