# Supplementary material for: Association between grandparent co-residence, socioeconomic status and dental caries among early school-aged children in Japan: A population-based prospective study
Source: Sci Rep. 2019 Aug 5;9:11345. doi: 10.1038/s41598-019-47730-3 (PMC6683122; doi:10.1038/s41598-019-47730-3)
Supplement: Supplementary file 1 — Supplementary figure and tables [file 41598_2019_47730_MOESM1_ESM.docx]

Association between grandparent co-residence, socioeconomic status and dental caries among early school-aged children in Japan: A population-based prospective study

Ayako Morita, Yusuke Matsuyama, Aya Isumi, Satomi Doi, Manami Ochi, Takeo Fujiwara

Supplementary Figure 1. Distribution of dft (grey) and DMFT (black) among the participants

| Supplementary Table 1. Outcome prevalence of teeth by tooth type and position by grandparent co-residence (n of individual teeth [level 1] = 80,897; n [level 2] = 3,578) | | | | | | | | | | | | | |
| --- | --- | --- | --- | --- | --- | --- | --- | --- | --- | --- | --- | --- | --- |
|  |  | Grandparent co-residence | | | | | | | | | | |  |
|  |  | No (n [level 1] = 3,247; n of individual teeth [level 2] = 73,596) | | | | |  | Yes (n [level 1] = 331; n of individual teeth [level 2] = 7,482) | | | | |  |
|  |  | Decidious teeth | | Permanent teeth | | Duplication of deciduous tooth and its permanent successor in the same position |  | Decidious teeth | | Permanent teeth | | Duplication of deciduous tooth and its permanent successor in the same position |  |
|  |  | decayed or filled | Sound | decayed, missing or filled | Sound |  |  | decayed or filled | Sound | decayed, missing or filled | Sound |  |  |
| All positions | | 5,035 | 39,187 | 320 | 29,055 | 166 |  | 596 | 3,935 | 38 | 2,913 | 15 |  |
|  | Upper central incisor | 94 | 1,073 | 5 | 4,819 | 31 |  | 6 | 119 | 0 | 475 | 0 |  |
|  | Upper lateral incisor | 79 | 3,491 | 3 | 1,741 | 22 |  | 13 | 363 | 0 | 165 | 1 |  |
|  | Upper canine | 97 | 6,268 | 0 | 7 | 1 |  | 13 | 638 | - | - | 0 |  |
|  | Upper first molar in deciduous teeth/first premolar in permanent teeth | 1,015 | 5,382 | 1 | 54 | 5 |  | 114 | 528 | 0 | 10 | 0 |  |
|  | Upper second molar in deciduous teeth/second premolar in permanent teeth | 884 | 5,562 | 0 | 9 | 2 |  | 109 | 550 | 0 | 2 | 2 |  |
|  | Upper first molar in permanent teeth | - | - | 104 | 5,356 | - |  | - | - | 12 | 542 | - |  |
|  | Upper second molar in permanent teeth | - | - | 0 | 0 | - |  | - | - | - | - | - |  |
|  | Lower central incisor | 0 | 170 | 0 | 6,266 | 24 |  | 0 | 22 | 0 | 630 | 3 |  |
|  | Lower laterlal incisor | 4 | 1,084 | 2 | 4,955 | 66 |  | 2 | 137 | 2 | 486 | 7 |  |
|  | Lower canine | 127 | 6,010 | 0 | 80 | 8 |  | 14 | 594 | 0 | 9 | 1 |  |
|  | Lower first molar in deciduous teeth/first premolar in permanent teeth | 1,556 | 4,874 | 0 | 24 | 6 |  | 180 | 470 | 0 | 1 | 1 |  |
|  | Lower second molar in deciduous teeth/second premolar in permanent teeth | 1,179 | 5,273 | 0 | 5 | 1 |  | 145 | 514 | - | - | 0 |  |
|  | Lower first molar in permanent teeth | - | - | 205 | 5,738 | - |  | - | - | 24 | 593 | - |  |
|  | Lower second molar in permanent teeth | - | - | - | - | - |  | - | - | - | - | - |  |

| Supplementary Table 2. Multilevel Poisson regression analysis of dental caries experience (decayed or filled primary teeth) (n of individual teeth [level 1] = 48,753; n [level 2] = 3,578) by grandparent co-residence | | | | | | | | | | | | | | | | | | | | | | | | | | | | | | | |
| --- | --- | --- | --- | --- | --- | --- | --- | --- | --- | --- | --- | --- | --- | --- | --- | --- | --- | --- | --- | --- | --- | --- | --- | --- | --- | --- | --- | --- | --- | --- | --- |
|  |  | Crude | | | | |  | Model I | | | | |  | Model II | | | | |  | Model III | | | | |  | Model IV | | | | |  |
| Outcome | | PR |  | 95%CI | | |  | APR |  | 95%CI | | |  | APR |  | 95%CI | | |  | APR |  | 95%CI | | |  | APR |  | 95%CI | | |  |
| Grandparent co-residence | |  |  |  |  |  |  |  |  |  |  |  |  |  |  |  |  |  |  |  |  |  |  |  |  |  |  |  |  |  |  |
|  | Yes | 1.29 | ( | 1.02 | - | 1.62 | ) | 1.30 | ( | 1.03 | - | 1.63 | ) | 1.15 | ( | 0.92 | - | 1.45 | ) | 1.13 | ( | 0.90 | - | 1.42 | ) | 1.06 | ( | 0.84 | - | 1.32 | ) |
|  | No | ref |  |  |  |  |  | ref |  |  |  |  |  | ref |  |  |  |  |  | ref |  |  |  |  |  | ref |  |  |  |  |  |
|  |  |  |  |  |  |  |  |  |  |  |  |  |  |  |  |  |  |  |  |  |  |  |  |  |  |  |  |  |  |  |  |
| Tooth position | |  |  |  |  |  |  |  |  |  |  |  |  |  |  |  |  |  |  |  |  |  |  |  |  |  |  |  |  |  |  |
|  | Upper central incisor | ref |  |  |  |  |  | ref |  |  |  |  |  | ref |  |  |  |  |  | ref |  |  |  |  |  | ref |  |  |  |  |  |
|  | Upper lateral incisor | 0.25 | ( | 0.19 | - | 0.34 | ) | 0.25 | ( | 0.19 | - | 0.34 | ) | 0.25 | ( | 0.19 | - | 0.34 | ) | 0.25 | ( | 0.19 | - | 0.34 | ) | 0.25 | ( | 0.19 | - | 0.34 | ) |
|  | Upper canine | 0.16 | ( | 0.12 | - | 0.21 | ) | 0.16 | ( | 0.12 | - | 0.21 | ) | 0.16 | ( | 0.12 | - | 0.21 | ) | 0.16 | ( | 0.12 | - | 0.21 | ) | 0.16 | ( | 0.12 | - | 0.21 | ) |
|  | Upper first molar | 1.64 | ( | 1.32 | - | 2.02 | ) | 1.64 | ( | 1.33 | - | 2.03 | ) | 1.64 | ( | 1.33 | - | 2.03 | ) | 1.65 | ( | 1.33 | - | 2.03 | ) | 1.65 | ( | 1.33 | - | 2.04 | ) |
|  | Upper second molar | 1.41 | ( | 1.14 | - | 1.74 | ) | 1.41 | ( | 1.14 | - | 1.75 | ) | 1.41 | ( | 1.14 | - | 1.74 | ) | 1.41 | ( | 1.14 | - | 1.75 | ) | 1.42 | ( | 1.14 | - | 1.75 | ) |
|  | Lower central incisor | 0.00 | ( | 0.00 | - | 0.00 | ) | 0.00 | ( | 0.00 | - | 0.00 | ) | 0.00 | ( | 0.00 | - | 0.00 | ) | 0.00 | ( | 0.00 | - | 0.00 | ) | 0.00 | ( | 0.00 | - | 0.00 | ) |
|  | Lower laterlal incisor | 0.06 | ( | 0.03 | - | 0.14 | ) | 0.06 | ( | 0.03 | - | 0.14 | ) | 0.06 | ( | 0.03 | - | 0.14 | ) | 0.06 | ( | 0.03 | - | 0.14 | ) | 0.06 | ( | 0.03 | - | 0.14 | ) |
|  | Lower canine | 0.21 | ( | 0.16 | - | 0.27 | ) | 0.21 | ( | 0.16 | - | 0.28 | ) | 0.21 | ( | 0.16 | - | 0.28 | ) | 0.21 | ( | 0.16 | - | 0.28 | ) | 0.21 | ( | 0.16 | - | 0.28 | ) |
|  | Lower first molar | 2.49 | ( | 2.02 | - | 3.06 | ) | 2.50 | ( | 2.02 | - | 3.08 | ) | 2.49 | ( | 2.02 | - | 3.07 | ) | 2.50 | ( | 2.03 | - | 3.08 | ) | 2.50 | ( | 2.03 | - | 3.08 | ) |
|  | Lower second molar | 1.88 | ( | 1.53 | - | 2.33 | ) | 1.89 | ( | 1.53 | - | 2.34 | ) | 1.89 | ( | 1.53 | - | 2.33 | ) | 1.89 | ( | 1.53 | - | 2.34 | ) | 1.90 | ( | 1.54 | - | 2.34 | ) |
|  |  |  |  |  |  |  |  |  |  |  |  |  |  |  |  |  |  |  |  |  |  |  |  |  |  |  |  |  |  |  |  |
| Sex | |  |  |  |  |  |  |  |  |  |  |  |  |  |  |  |  |  |  |  |  |  |  |  |  |  |  |  |  |  |  |
|  | Male | ref |  |  |  |  |  | ref |  |  |  |  |  | ref |  |  |  |  |  | ref |  |  |  |  |  | ref |  |  |  |  |  |
|  | Female | 0.88 | ( | 0.77 | - | 1.01 | ) | 0.85 | ( | 0.74 | - | 0.98 | ) | 0.84 | ( | 0.74 | - | 0.97 | ) | 0.82 | ( | 0.72 | - | 0.94 | ) | 0.85 | ( | 0.74 | - | 0.97 | ) |
|  |  |  |  |  |  |  |  |  |  |  |  |  |  |  |  |  |  |  |  |  |  |  |  |  |  |  |  |  |  |  |  |
| Age (month) | | 1.00 | ( | 0.98 | - | 1.02 | ) | 1.00 | ( | 0.98 | - | 1.02 | ) | 1.00 | ( | 0.98 | - | 1.02 | ) | 1.00 | ( | 0.98 | - | 1.02 | ) | 0.99 | ( | 0.97 | - | 1.01 | ) |
|  |  |  |  |  |  |  |  |  |  |  |  |  |  |  |  |  |  |  |  |  |  |  |  |  |  |  |  |  |  |  |  |
| Maternal education | |  |  |  |  |  |  |  |  |  |  |  |  |  |  |  |  |  |  |  |  |  |  |  |  |  |  |  |  |  |  |
|  | Highschool graduate or less | ref |  |  |  |  |  |  |  |  |  |  |  | ref |  |  |  |  |  | ref |  |  |  |  |  | ref |  |  |  |  |  |
|  | Some college | 0.72 | ( | 0.61 | - | 0.84 | ) |  |  |  |  |  |  | 0.77 | ( | 0.66 | - | 0.90 | ) | 0.80 | ( | 0.69 | - | 0.94 | ) | 0.90 | ( | 0.77 | - | 1.05 | ) |
|  | College or University graduate | 0.46 | ( | 0.38 | - | 0.56 | ) |  |  |  |  |  |  | 0.52 | ( | 0.43 | - | 0.64 | ) | 0.57 | ( | 0.46 | - | 0.69 | ) | 0.66 | ( | 0.54 | - | 0.81 | ) |
|  | Other | 0.61 | ( | 0.25 | - | 1.49 | ) |  |  |  |  |  |  | 0.61 | ( | 0.25 | - | 1.47 | ) | 0.58 | ( | 0.24 | - | 1.41 | ) | 0.65 | ( | 0.27 | - | 1.55 | ) |
|  |  |  |  |  |  |  |  |  |  |  |  |  |  |  |  |  |  |  |  |  |  |  |  |  |  |  |  |  |  |  |  |
| Household income (yen) | |  |  |  |  |  |  |  |  |  |  |  |  |  |  |  |  |  |  |  |  |  |  |  |  |  |  |  |  |  |  |
|  | <3 million | ref |  |  |  |  |  |  |  |  |  |  |  | ref |  |  |  |  |  | ref |  |  |  |  |  | ref |  |  |  |  |  |
|  | 3 million ~ <6 million | 0.73 | ( | 0.58 | - | 0.92 | ) |  |  |  |  |  |  | 0.85 | ( | 0.66 | - | 1.09 | ) | 0.83 | ( | 0.65 | - | 1.06 | ) | 0.86 | ( | 0.68 | - | 1.10 | ) |
|  | 6 million ~ < 10 million | 0.49 | ( | 0.39 | - | 0.62 | ) |  |  |  |  |  |  | 0.63 | ( | 0.48 | - | 0.82 | ) | 0.61 | ( | 0.47 | - | 0.79 | ) | 0.66 | ( | 0.51 | - | 0.86 | ) |
|  | 10 million and above | 0.62 | ( | 0.45 | - | 0.84 | ) |  |  |  |  |  |  | 0.86 | ( | 0.62 | - | 1.20 | ) | 0.81 | ( | 0.58 | - | 1.12 | ) | 0.88 | ( | 0.63 | - | 1.21 | ) |
|  |  |  |  |  |  |  |  |  |  |  |  |  |  |  |  |  |  |  |  |  |  |  |  |  |  |  |  |  |  |  |  |
| Living arrangement with parents | |  |  |  |  |  |  |  |  |  |  |  |  |  |  |  |  |  |  |  |  |  |  |  |  |  |  |  |  |  |  |
|  | Living with two parents | ref |  |  |  |  |  |  |  |  |  |  |  | ref |  |  |  |  |  | ref |  |  |  |  |  | ref |  |  |  |  |  |
|  | Lone-parent household | 1.67 | ( | 1.32 | - | 2.10 | ) |  |  |  |  |  |  | 1.23 | ( | 0.94 | - | 1.60 | ) | 1.25 | ( | 0.96 | - | 1.62 | ) | 1.23 | ( | 0.95 | - | 1.60 | ) |
|  |  |  |  |  |  |  |  |  |  |  |  |  |  |  |  |  |  |  |  |  |  |  |  |  |  |  |  |  |  |  |  |
| Birth order | |  |  |  |  |  |  |  |  |  |  |  |  |  |  |  |  |  |  |  |  |  |  |  |  |  |  |  |  |  |  |
|  | First-born or only child | ref |  |  |  |  |  |  |  |  |  |  |  |  |  |  |  |  |  | ref |  |  |  |  |  | ref |  |  |  |  |  |
|  | Middle or last-born | 1.75 | ( | 1.53 | - | 2.01 | ) |  |  |  |  |  |  |  |  |  |  |  |  | 1.70 | ( | 1.49 | - | 1.95 | ) | 1.59 | ( | 1.39 | - | 1.82 | ) |
|  |  |  |  |  |  |  |  |  |  |  |  |  |  |  |  |  |  |  |  |  |  |  |  |  |  |  |  |  |  |  |  |
| Snacking habits | |  |  |  |  |  |  |  |  |  |  |  |  |  |  |  |  |  |  |  |  |  |  |  |  |  |  |  |  |  |  |
|  | Conrolled by parent(s) | ref |  |  |  |  |  |  |  |  |  |  |  |  |  |  |  |  |  |  |  |  |  |  |  | ref |  |  |  |  |  |
|  | Eating at any time | 2.06 | ( | 1.76 | - | 2.40 | ) |  |  |  |  |  |  |  |  |  |  |  |  |  |  |  |  |  |  | 1.57 | ( | 1.35 | - | 1.84 | ) |
|  |  |  |  |  |  |  |  |  |  |  |  |  |  |  |  |  |  |  |  |  |  |  |  |  |  |  |  |  |  |  |  |
| Sugar-sweetened beverage intake | |  |  |  |  |  |  |  |  |  |  |  |  |  |  |  |  |  |  |  |  |  |  |  |  |  |  |  |  |  |  |
|  | <1 time/day | ref |  |  |  |  |  |  |  |  |  |  |  |  |  |  |  |  |  |  |  |  |  |  |  | ref |  |  |  |  |  |
|  | 1 time or more/day | 1.99 | ( | 1.69 | - | 2.36 | ) |  |  |  |  |  |  |  |  |  |  |  |  |  |  |  |  |  |  | 1.61 | ( | 1.37 | - | 1.90 | ) |
|  |  |  |  |  |  |  |  |  |  |  |  |  |  |  |  |  |  |  |  |  |  |  |  |  |  |  |  |  |  |  |  |
| Teethbrushing habits | |  |  |  |  |  |  |  |  |  |  |  |  |  |  |  |  |  |  |  |  |  |  |  |  |  |  |  |  |  |  |
|  | <2 times/day | ref |  |  |  |  |  |  |  |  |  |  |  |  |  |  |  |  |  |  |  |  |  |  |  | ref |  |  |  |  |  |
|  | 2 times or more/day | 0.62 | ( | 0.53 | - | 0.73 | ) |  |  |  |  |  |  |  |  |  |  |  |  |  |  |  |  |  |  | 0.77 | ( | 0.66 | - | 0.90 | ) |
|  |  |  |  |  |  |  |  |  |  |  |  |  |  |  |  |  |  |  |  |  |  |  |  |  |  |  |  |  |  |  |  |
| Teethbrusihng supervision | |  |  |  |  |  |  |  |  |  |  |  |  |  |  |  |  |  |  |  |  |  |  |  |  |  |  |  |  |  |  |
|  | No | ref |  |  |  |  |  |  |  |  |  |  |  |  |  |  |  |  |  |  |  |  |  |  |  | ref |  |  |  |  |  |
|  | Yes | 0.60 | ( | 0.44 | - | 0.71 | ) |  |  |  |  |  |  |  |  |  |  |  |  |  |  |  |  |  |  | 0.83 | ( | 0.69 | - | 1.00 | ) |
| PR = Prevalence Ratio; APR = Adjusted Prevalence Ratio (Model I adjusted for tooth type and demographics; Model II further adjusted for parental SES; Model III further adjusted for birth order; Model IV further adjusted for health behaviors) | | | | | | | | | | | | | | | | | | | | | | | | | | | | | | | |

| Supplementary Table 3. Multilevel Poisson regression analysis of dental caries experience (Decayed, Missing, or Filled permanent teeth) (n of individual teeth [level 1] = 32,325; n [level 2] = 3,578) by grandparent co-residence | | | | | | | | | | | | | | | | | | | | | | | | | | | | | | | |
| --- | --- | --- | --- | --- | --- | --- | --- | --- | --- | --- | --- | --- | --- | --- | --- | --- | --- | --- | --- | --- | --- | --- | --- | --- | --- | --- | --- | --- | --- | --- | --- |
|  |  | Crude | | | | |  | Model I | | | | |  | Model II | | | | |  | Model III | | | | |  | Model IV | | | | |  |
|  |  |  |  |  |  |  |  |  |  |  |  |  |  |  |  |  |  |  |  |  |  |  |  |  |  |  |  |  |  |  |  |
| Outcome | | PR |  | 95%CI | | |  | APR |  | 95%CI | | |  | APR |  | 95%CI | | |  | APR |  | 95%CI | | |  | APR |  | 95%CI | | |  |
| Grandparent co-residence | |  |  |  |  |  |  |  |  |  |  |  |  |  |  |  |  |  |  |  |  |  |  |  |  |  |  |  |  |  |  |
|  | Yes | 1.22 | ( | 0.72 | - | 2.05 | ) | 1.17 | ( | 0.69 | - | 1.99 | ) | 1.15 | ( | 0.67 | - | 1.97 | ) | 1.11 | ( | 0.65 | - | 1.92 | ) | 1.06 | ( | 0.62 | - | 1.83 | ) |
|  | No | ref |  |  |  |  |  | ref |  |  |  |  |  | ref |  |  |  |  |  | ref |  |  |  |  |  | ref |  |  |  |  |  |
|  |  |  |  |  |  |  |  |  |  |  |  |  |  |  |  |  |  |  |  |  |  |  |  |  |  |  |  |  |  |  |  |
| Teeth position | |  |  |  |  |  |  |  |  |  |  |  |  |  |  |  |  |  |  |  |  |  |  |  |  |  |  |  |  |  |  |
|  | Upper central incisor | ref |  |  |  |  |  | ref |  |  |  |  |  | ref |  |  |  |  |  | ref |  |  |  |  |  | ref |  |  |  |  |  |
|  | Upper lateral incisor | 1.48 | ( | 0.35 | - | 3.00 | ) | 1.47 | ( | 0.35 | - | 6.19 | ) | 1.47 | ( | 0.35 | - | 6.18 | ) | 1.48 | ( | 0.35 | - | 6.20 | ) | 1.48 | ( | 0.35 | - | 6.22 | ) |
|  | Upper canine | 0.00 | ( | 0.00 | - | 0.00 | ) | 0.00 | ( | 0.00 | - | 0.00 | ) | 0.00 | ( | 0.00 | - | 0.00 | ) | 0.00 | ( | 0.00 | - | 0.00 | ) | 0.00 | ( | 0.00 | - | 0.00 | ) |
|  | Upper first premolar | 8.36 | ( | 0.92 | - | 75.71 | ) | 8.37 | ( | 0.92 | - | 75.81 | ) | 8.43 | ( | 0.93 | - | 76.50 | ) | 8.24 | ( | 0.91 | - | 74.65 | ) | 8.21 | ( | 0.91 | - | 74.32 | ) |
|  | Upper second premolar | 0.00 | ( | 0.00 | - | 0.00 | ) | 0.00 | ( | 0.00 | - | 0.00 | ) | 0.00 | ( | 0.00 | - | 0.00 | ) | 0.00 | ( | 0.00 | - | 0.00 | ) | 0.00 | ( | 0.00 | - | 0.00 | ) |
|  | Upper first molar | 21.09 | ( | 8.61 |  | 51.68 | ) | 21.13 | ( | 8.62 | - | 51.77 | ) | 21.12 | ( | 8.62 | - | 51.74 | ) | 21.12 | ( | 8.62 | - | 51.75 | ) | 21.10 | ( | 8.61 | - | 51.70 | ) |
|  | Upper second molar | 0.00 | ( | 0.00 | - | 0.00 | ) | 0.00 | ( | 0.00 | - | 0.00 | ) | 0.00 | ( | 0.00 | - | 0.00 | ) | 0.00 | ( | 0.00 | - | 0.00 | ) | 0.00 | ( | 0.00 | - | 0.00 | ) |
|  | Lower central incisor | 0.00 | ( | 0.00 | - | 0.00 | ) | 0.00 | ( | 0.00 | - | 0.00 | ) | 0.00 | ( | 0.00 | - | 0.00 | ) | 0.00 | ( | 0.00 | - | 0.00 | ) | 0.00 | ( | 0.00 | - | 0.00 | ) |
|  | Lower laterlal incisor | 0.78 | ( | 0.21 | - | 2.91 | ) | 0.78 | ( | 0.21 | - | 2.91 | ) | 0.78 | ( | 0.21 | - | 2.91 | ) | 0.78 | ( | 0.21 | - | 2.90 | ) | 0.78 | ( | 0.21 | - | 2.90 | ) |
|  | Lower canine | 0.00 | ( | 0.00 | - | 0.00 | ) | 0.00 | ( | 0.00 | - | 0.00 | ) | 0.00 | ( | 0.00 | - | 0.00 | ) | 0.00 | ( | 0.00 | - | 0.00 | ) | 0.00 | ( | 0.00 | - | 0.00 | ) |
|  | Lower first premolar | 0.00 | ( | 0.00 | - | 0.00 | ) | 0.00 | ( | 0.00 | - | 0.00 | ) | 0.00 | ( | 0.00 | - | 0.00 | ) | 0.00 | ( | 0.00 | - | 0.00 | ) | 0.00 | ( | 0.00 | - | 0.00 | ) |
|  | Lower second premolar | 0.00 | ( | 0.00 | - | 0.00 | ) | 0.00 | ( | 0.00 | - | 0.00 | ) | 0.00 | ( | 0.00 | - | 0.00 | ) | 0.00 | ( | 0.00 | - | 0.00 | ) | 0.00 | ( | 0.00 | - | 0.00 | ) |
|  | Lower first molar | 38.83 | ( | 15.99 | - | 94.26 | ) | 38.87 | ( | 16.01 | - | 94.36 | ) | 38.89 | ( | 16.02 | - | 94.41 | ) | 38.89 | ( | 16.02 | - | 94.42 | ) | 38.90 | ( | 16.02 | - | 94.43 | ) |
|  | Lower second molar | - |  | - |  | - |  | - |  | - |  | - |  | - |  | - |  | - |  | - |  | - |  | - |  | - |  | - |  | - |  |
|  |  |  |  |  |  |  |  |  |  |  |  |  |  |  |  |  |  |  |  |  |  |  |  |  |  |  |  |  |  |  |  |
| Sex | |  |  |  |  |  |  |  |  |  |  |  |  |  |  |  |  |  |  |  |  |  |  |  |  |  |  |  |  |  |  |
|  | Male | ref |  |  |  |  |  | ref |  |  |  |  |  | ref |  |  |  |  |  | ref |  |  |  |  |  | ref |  |  |  |  |  |
|  | Female | 1.13 | ( | 0.82 | - | 1.55 | ) | 1.19 | ( | 0.86 | - | 1.63 | ) | 1.16 | ( | 0.84 | - | 1.59 | ) | 1.13 | ( | 0.82 | - | 1.55 | ) | 1.17 | ( | 0.85 | - | 1.61 | ) |
|  |  |  |  |  |  |  |  |  |  |  |  |  |  |  |  |  |  |  |  |  |  |  |  |  |  |  |  |  |  |  |  |
| Age (month) | | 1.01 | ( | 0.96 | - | 1.05 | ) | 1.01 | ( | 0.97 | - | 1.05 | ) | 1.01 | ( | 0.97 | - | 1.06 | ) | 1.01 | ( | 0.97 | - | 1.06 | ) | 1.01 | ( | 0.96 | - | 1.05 | ) |
|  |  |  |  |  |  |  |  |  |  |  |  |  |  |  |  |  |  |  |  |  |  |  |  |  |  |  |  |  |  |  |  |
| Maternal education | |  |  |  |  |  |  |  |  |  |  |  |  |  |  |  |  |  |  |  |  |  |  |  |  |  |  |  |  |  |  |
|  | Junior high school graduate | ref |  |  |  |  |  |  |  |  |  |  |  | ref |  |  |  |  |  | ref |  |  |  |  |  | ref |  |  |  |  |  |
|  | Highschool graduate | 0.94 | ( | 0.66 | - | 1.33 | ) |  |  |  |  |  |  | 0.93 | ( | 0.65 | - | 1.34 | ) | 0.97 | ( | 0.67 | - | 1.39 | ) | 1.01 | ( | 0.70 | - | 1.46 | ) |
|  | Some college | 0.71 | ( | 0.45 | - | 1.12 | ) |  |  |  |  |  |  | 0.71 | ( | 0.44 | - | 1.15 | ) | 0.80 | ( | 0.49 | - | 1.29 | ) | 0.87 | ( | 0.53 | - | 1.42 | ) |
|  | College or University graduate | 4.25 | ( | 1.12 | - | 16.10 | ) |  |  |  |  |  |  | 4.12 | ( | 1.07 | - | 15.90 | ) | 3.86 | ( | 0.99 | - | 15.08 | ) | 4.16 | ( | 1.07 | - | 16.24 | ) |
|  | Other | 0.57 | ( | 0.15 | - | 2.19 | ) |  |  |  |  |  |  | 0.56 | ( | 0.14 | - | 2.26 | ) | 0.58 | ( | 0.15 | - | 2.34 | ) | 0.63 | ( | 0.16 | - | 2.56 | ) |
|  |  |  |  |  |  |  |  |  |  |  |  |  |  |  |  |  |  |  |  |  |  |  |  |  |  |  |  |  |  |  |  |
| Household income (yen) | |  |  |  |  |  |  |  |  |  |  |  |  |  |  |  |  |  |  |  |  |  |  |  |  |  |  |  |  |  |  |
|  | <3 million | ref |  |  |  |  |  |  |  |  |  |  |  | ref |  |  |  |  |  | ref |  |  |  |  |  | ref |  |  |  |  |  |
|  | 3 million ~ <6 million | 0.93 | ( | 0.54 | - | 1.59 | ) |  |  |  |  |  |  | 0.87 | ( | 0.47 | - | 1.59 | ) | 0.84 | ( | 0.46 | - | 1.55 | ) | 0.85 | ( | 0.46 | - | 1.57 | ) |
|  | 6 million ~ < 10 million | 0.83 | ( | 0.47 | - | 1.45 | ) |  |  |  |  |  |  | 0.80 | ( | 0.42 | - | 1.52 | ) | 0.76 | ( | 0.40 | - | 1.44 | ) | 0.78 | ( | 0.41 | - | 1.48 | ) |
|  | 10 million and above | 0.89 | ( | 0.44 | - | 1.83 | ) |  |  |  |  |  |  | 0.94 | ( | 0.42 | - | 2.08 | ) | 0.86 | ( | 0.39 | - | 1.92 | ) | 0.90 | ( | 0.40 | - | 1.99 | ) |
|  |  |  |  |  |  |  |  |  |  |  |  |  |  |  |  |  |  |  |  |  |  |  |  |  |  |  |  |  |  |  |  |
| Living arrangement with parents | |  |  |  |  |  |  |  |  |  |  |  |  |  |  |  |  |  |  |  |  |  |  |  |  |  |  |  |  |  |  |
|  | Living with two parents | ref |  |  |  |  |  |  |  |  |  |  |  | ref |  |  |  |  |  | ref |  |  |  |  |  | ref |  |  |  |  |  |
|  | Lone-parent household | 0.94 | ( | 0.53 | - | 1.66 | ) |  |  |  |  |  |  | 0.77 | ( | 0.39 | - | 1.50 | ) | 0.78 | ( | 0.40 | - | 1.52 | ) | 0.78 | ( | 0.40 | - | 1.52 | ) |
|  |  |  |  |  |  |  |  |  |  |  |  |  |  |  |  |  |  |  |  |  |  |  |  |  |  |  |  |  |  |  |  |
| Birth order | |  |  |  |  |  |  |  |  |  |  |  |  |  |  |  |  |  |  |  |  |  |  |  |  |  |  |  |  |  |  |
|  | First-born or only child | ref |  |  |  |  |  |  |  |  |  |  |  |  |  |  |  |  |  | ref |  |  |  |  |  | ref |  |  |  |  |  |
|  | Middle or last-born | 1.77 | ( | 1.29 | - | 2.44 | ) |  |  |  |  |  |  |  |  |  |  |  |  | 1.71 | ( | 1.23 | - | 2.38 | ) | 1.67 | ( | 1.20 | - | 2.33 | ) |
|  |  |  |  |  |  |  |  |  |  |  |  |  |  |  |  |  |  |  |  |  |  |  |  |  |  |  |  |  |  |  |  |
| Snacking habits | |  |  |  |  |  |  |  |  |  |  |  |  |  |  |  |  |  |  |  |  |  |  |  |  |  |  |  |  |  |  |
|  | Conrolled by parent(s) | ref |  |  |  |  |  |  |  |  |  |  |  |  |  |  |  |  |  |  |  |  |  |  |  | ref |  |  |  |  |  |
|  | Eating at any time | 1.49 | ( | 1.05 | - | 2.11 | ) |  |  |  |  |  |  |  |  |  |  |  |  |  |  |  |  |  |  | 1.29 | ( | 0.88 | - | 1.87 | ) |
|  |  |  |  |  |  |  |  |  |  |  |  |  |  |  |  |  |  |  |  |  |  |  |  |  |  |  |  |  |  |  |  |
| Sugar-sweetened beverage intake | |  |  |  |  |  |  |  |  |  |  |  |  |  |  |  |  |  |  |  |  |  |  |  |  |  |  |  |  |  |  |
|  | <1 time/day | ref |  |  |  |  |  |  |  |  |  |  |  |  |  |  |  |  |  |  |  |  |  |  |  | ref |  |  |  |  |  |
|  | 1 time or more/day | 1.35 | ( | 0.92 | - | 1.98 | ) |  |  |  |  |  |  |  |  |  |  |  |  |  |  |  |  |  |  | 1.24 | ( | 0.83 | - | 1.84 | ) |
|  |  |  |  |  |  |  |  |  |  |  |  |  |  |  |  |  |  |  |  |  |  |  |  |  |  |  |  |  |  |  |  |
| Teethbrushing habits | |  |  |  |  |  |  |  |  |  |  |  |  |  |  |  |  |  |  |  |  |  |  |  |  |  |  |  |  |  |  |
|  | <2 times/day | ref |  |  |  |  |  |  |  |  |  |  |  |  |  |  |  |  |  |  |  |  |  |  |  | ref |  |  |  |  |  |
|  | 2 times or more/day | 0.68 | ( | 0.48 | - | 0.98 | ) |  |  |  |  |  |  |  |  |  |  |  |  |  |  |  |  |  |  | 0.77 | ( | 0.53 | - | 1.12 | ) |
|  |  |  |  |  |  |  |  |  |  |  |  |  |  |  |  |  |  |  |  |  |  |  |  |  |  |  |  |  |  |  |  |
| Teethbrusihng supervision | |  |  |  |  |  |  |  |  |  |  |  |  |  |  |  |  |  |  |  |  |  |  |  |  |  |  |  |  |  |  |
|  | No | ref |  |  |  |  |  |  |  |  |  |  |  |  |  |  |  |  |  |  |  |  |  |  |  | ref |  |  |  |  |  |
|  | Yes | 0.64 | ( | 0.63 | - | 1.56 | ) |  |  |  |  |  |  |  |  |  |  |  |  |  |  |  |  |  |  | 1.19 | ( | 0.75 | - | 1.88 | ) |
| PR = Prevalence Ratio; APR = Adjusted Prevalence Ratio (Model I adjusted for tooth type and demographics; Model II further adjusted for parental SES; Model III further adjusted for birth order; Model IV further adjusted for health behaviors) | | | | | | | | | | | | | | | | | | | | | | | | | | | | | | | |
